# ATTACKED BY BOTS AND HACKED BY BAD APPLES: LESSONS LEARNED NAVIGATING RESEARCH PARTICIPANT FRAUD

**DOI:** 10.1093/geroni/igad104.1177

**Published:** 2023-12-21

**Authors:** Erica Frechman, Harleah Buck, Cathy Maxwell

**Affiliations:** Atrium Health Wake Forest Baptist, Winston-Salem, North Carolina, United States; University of Iowa, Iowa City, Iowa, United States; Vanderbilt University, Nashville, Tennessee, United States

## Abstract

In a time of emerging technologies for participant recruitment and data collection, researchers need proven and comprehensive strategies to protect research integrity. Incorporation of proactive, rather than reactive, methods to prevent fraudulence will mitigate additional stressors and headaches associated with fraud and fraud detection. This session presents a case which describes a researcher’s experience detecting, navigating, problem-solving, and then managing participant fraud that occurred twice in a multi-methods study. Participants were recruited from a senior center and two YMCA sites using social media platforms and email distribution lists which linked them with an online survey. After the initial study launch, a “bot attack” with “bot fast” deception within the study survey caused the study to shut down. The researcher worked with the study team, community sites, and IRB to detect potential causes of fraud and implement protective factors to ensure quality control and deter the bot system. Unfortunately, after the second study launch, the survey was once again successfully attacked. This time the researcher chose to work with the study team in order to create a step-wise rigorous approach to verifying accurate data and participants, while working to maintain trust of the community sites and real participants. Finally, to mitigate further fraud, the researcher pivoted to the use of ResearchMatch to ensure recruitment goals and trustworthy data. Within this presentation, the researcher will share lessons learned in survey fraud, proactive strategies to prevent fraudulence, communication methods interacting with IRB, study sites, and participants, and maneuvering perils involved in manuscript preparation.

